# Clinician and Patient Perspectives on the Use of Passive Mobile Monitoring and Self-Tracking for Patients With Serious Mental Illness: User-Centered Approach

**DOI:** 10.2196/46909

**Published:** 2023-10-24

**Authors:** Melissa Medich, Shay L Cannedy, Lauren C Hoffmann, Melissa Y Chinchilla, Jose M Pila, Stephanie A Chassman, Ronald A Calderon, Alexander S Young

**Affiliations:** 1 Center for the Study of Healthcare Innovation, Implementation and Policy VA Greater Los Angeles Healthcare System U.S. Department of Veteran Affairs North Hills, CA United States; 2 The Lundquist Institute for Biomedical Research Torrance, CA United States; 3 Mental Illness Research, Education and Clinical Center VA Greater Los Angeles Healthcare System U.S. Department of Veteran Affairs Los Angeles, CA United States; 4 Department of Psychiatry and Biobehavioral Sciences Semel Institute for Neuroscience and Human Behavior University California Los Angeles Geffen School of Medicine Los Angeles, CA United States

**Keywords:** serious mental illness, mobile health, mental health, passive sensing, health informatics, behavior, self-tracking, monitoring, mental illness, prevention, acceptability, usability, usefulness, application, tool, management, mobile phone

## Abstract

**Background:**

Early intervention in mental health crises can prevent negative outcomes. A promising new direction is remote mental health monitoring using smartphone technology to passively collect data from individuals to rapidly detect the worsening of serious mental illness (SMI). This technology may benefit patients with SMI, but little is known about health IT acceptability among this population or their mental health clinicians.

**Objective:**

We used the Health Information Technology Acceptability Model to analyze the acceptability and usability of passive mobile monitoring and self-tracking among patients with serious mental illness and their mental health clinicians.

**Methods:**

Data collection took place between December 2020 and June 2021 in 1 Veterans Administration health care system. Interviews with mental health clinicians (n=16) assessed the acceptability of mobile sensing, its usefulness as a tool to improve clinical assessment and care, and recommendations for program refinements. Focus groups with patients with SMI (n=3 groups) and individual usability tests (n=8) elucidated patient attitudes about engaging in health IT and perceptions of its usefulness as a tool for self-tracking and improving mental health assessments.

**Results:**

Clinicians discussed the utility of web-based data dashboards to monitor patients with SMI health behaviors and receiving alerts about their worsening health. Potential benefits included improving clinical care, capturing behaviors patients do not self-report, watching trends, and receiving alerts. Clinicians’ concerns included increased workloads tied to dashboard data review, lack of experience using health IT in clinical care, and how SMI patients’ associated paranoia and financial instability would impact patient uptake. Despite concerns, all mental health clinicians stated that they would recommend it. Almost all patients with SMI were receptive to using smartphone dashboards for self-monitoring and having behavioral change alerts sent to their mental health clinicians. They found the mobile app easy to navigate and dashboards easy to find and understand. Patient concerns centered on privacy and “government tracking,” and their phone’s battery life and data plans. Despite concerns, most reported that they would use it.

**Conclusions:**

Many people with SMI would like to have mobile informatics tools that can support their illness and recovery. Similar to other populations (eg, older adults, people experiencing homelessness) this population presents challenges to adoption and implementation. Health care organizations will need to provide resources to address these and support successful illness management. Clinicians are supportive of technological approaches, with adapting informatics data into their workflow as the primary challenge. Despite clear challenges, technological developments are increasingly designed to be acceptable to patients. The research development–clinical deployment gap must be addressed by health care systems, similar to computerized cognitive training. It will ensure clinicians operate at the top of their skill set and are not overwhelmed by administrative tasks, data summarization, or reviewing data that do not indicate a need for intervention.

**International Registered Report Identifier (IRRID):**

RR2-10.2196/39010

## Introduction

Serious mental illnesses, such as schizophrenia and bipolar disorder, are conditions that result in poor outcomes when not appropriately treated. These illnesses are challenging to treat and usually require years of monitoring and adjustments in treatment [1-3]. Stress, substance misuse, or incomplete medication adherence can cause rapid worsening of symptoms, with consequences that can include job loss, homelessness, suicide, incarceration, or hospitalization. Treatment visits are relatively infrequent. Thus, illness exacerbations usually occur with no clinician awareness, leaving little opportunity to make treatment adjustments [4,5]. Tools are needed that quickly detect worsening illness and improve quality of care.

Computerized assessments have been used for years with patients who have serious mental illness (SMI) [6,7]. Regarding the collection of mobile data, studies in bipolar disorder found that depressive and manic symptoms correlated with activity and phone communication [8,9]. Other studies found that activity, movement, and location were associated with mood states in bipolar disorder [4,10]. Studies in schizophrenia have monitored indicators of activity, communication, and sleep. In 1 study, 95% of patients were comfortable with sensing and two-thirds did not have privacy concerns [11]. In SMI, researchers have found associations between stress, depression, psychotic experiences, and sensor data related to sleep, activity, and communication [12,13]; and associations between hospitalization, outpatient use, location, activity, communication, and screen use [2].

Mobile devices could be used to detect the worsening of psychiatric illness and improve care [8,12,14-16]. The majority of people with SMI use smartphones [6,17]. These phones generate substantial passive data from numerous sensors that researchers have used to estimate mental health status and behaviors [2,10,12,18-20]. However, efforts to use mobile technologies in this population have encountered challenges related to usability and design [6,21-23]. People with SMI often have cognitive deficits, persistent psychiatric symptoms, and social and economic disadvantages [1,20,22]. It is not known whether patients in usual care systems will engage in mobile interventions that include monitoring of their data. It is also not clear how to design smartphone monitoring systems that are feasible and useful for patients with SMI and their clinicians.

**Figure 1:**
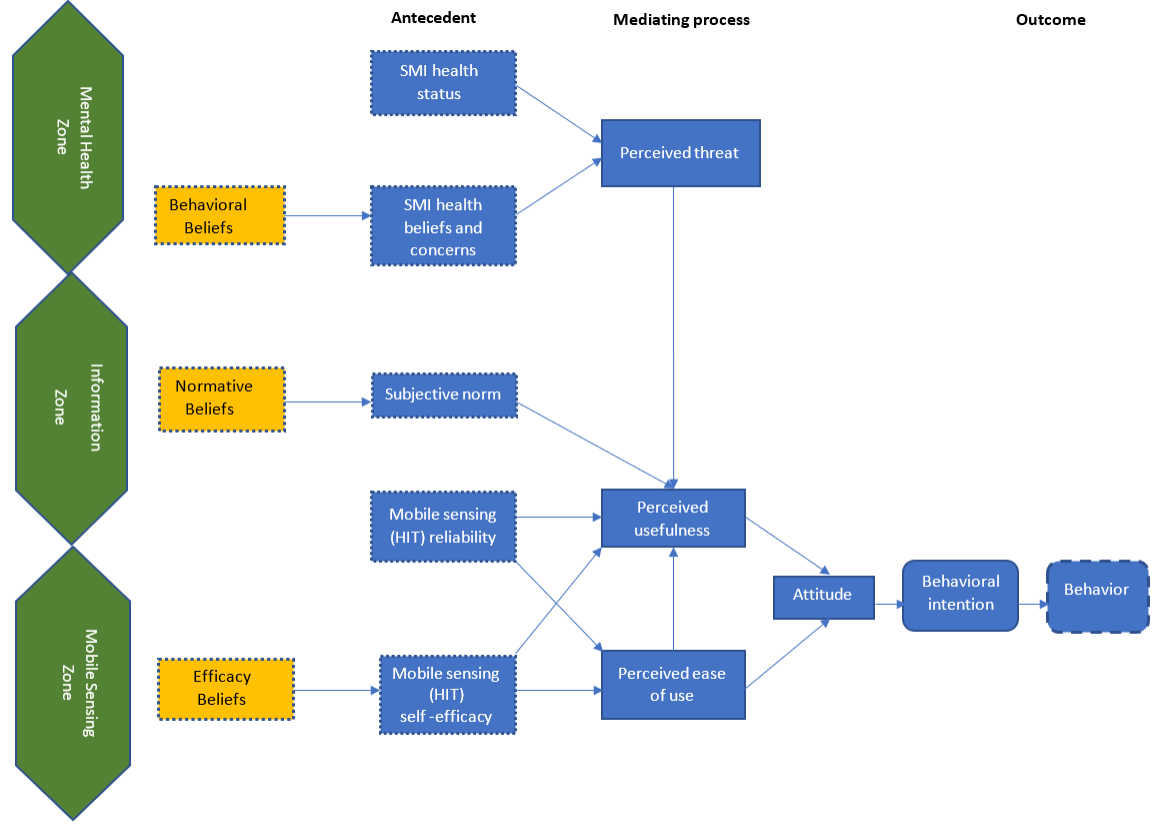
The structure of Health Information Technology Acceptance Model adopted for Mobile Sensing. HIT: health information technology; SMI: serious mental illness.

The Health Information Technology Acceptance Model (HITAM, Figure 1) is useful for qualitatively studying the acceptability and usability of mobile apps. HITAM integrates the Technology Acceptance Model [24] with key concepts of the Health Belief Model, one of the most widely used models for understanding health behaviors and identifying health beliefs [25]. HITAM explains how factors (eg, health status and beliefs, subjective norms, technology reliability, and self-efficacy) influence interactions with health information technology (HIT), such as the Mobile Sensing app. The framework considers behavioral, normative, and efficacy beliefs to lead to the concepts of perceived threat, perceived usefulness, and ease of use, respectively. The HITAM framework has been adapted in qualitative studies of user experiences to mobile phone app usage for chronic illnesses such as the self-management of type 2 diabetes [26,27] and the value, usability, and functionality during the development of a quality-of-life assessment app for people with SMI [28]. Application of HITAM to the concept of passive mobile sensing can enhance our understanding of how mobile apps could form behavioral intentions around passive mobile sensing for patients with SMI.

This study investigates patient and clinician perspectives, and the acceptability and usability of passive mobile monitoring designed to detect and predict worsening symptoms, with the goals of facilitating earlier assessment, timely intervention, and improved outcomes. This study informs intervention development and mobile app usability and seeks to maximize adoption and engagement through user-centered design in people with SMI. It is possible that passive mobile sensing via the Mobile Sensing app could empower patients with SMI to self-monitor their symptoms and behaviors. For mental health care clinicians, the integration of such apps into care may further improve patient outcomes. This study investigates patient and clinician perspectives, and the acceptability and usability of passive mobile monitoring designed to detect and predict worsening symptoms, with the goals of facilitating earlier assessment, timely intervention, and improved outcomes.

## Methods

### Ethics Approval

The study has been approved by the Institutional Review Board of the VA Greater Los Angeles (1615834-20).

### Study Design

This study was conducted as part of research developing a mobile sensing informatics intervention and conducting pilot use of the intervention in a population with SMI. The protocol, including the details of the methods for this study, has been previously published [16]. Patient focus groups and usability tests were conducted to determine the acceptability and usability of the Mobile Sensing app for self-monitoring while clinician interviews determined the acceptability of the mobile sensing data to improve clinical care. Data were collected during the preimplementation user-centered design phase of the passive mobile sensing study (October 2020 to June 2021). This focused on usability and perceptions regarding the mobile app and informed modifications to the product during phase 1 of the mobile sensing study design (user-centered design phase) using patient focus groups and usability tests to inform mobile app modifications for phase 2, the mobile sensing phase of the study [16]. Results from this study contribute to the field of passive mobile sensing technologies and self-tracking in patients with SMI to improve clinical care and patient outcomes.

### Design of the Mobile Sensing App Prototype

**Figure 2:**
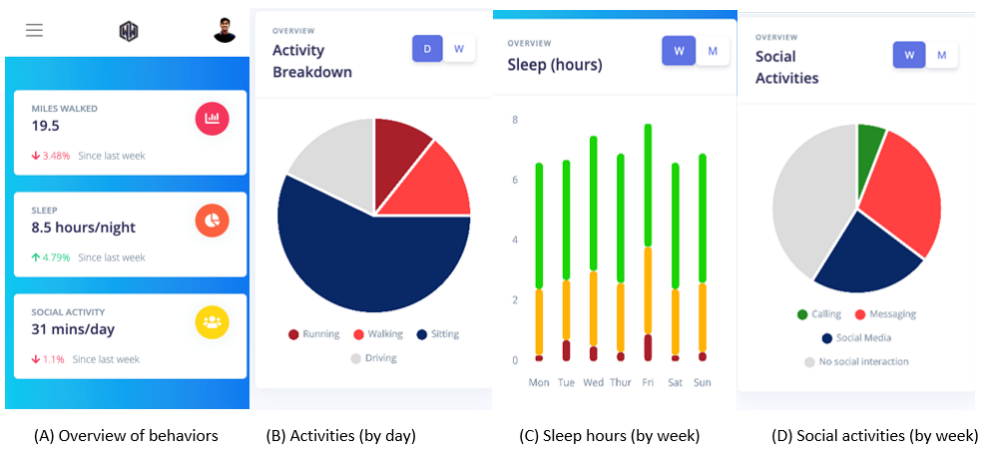
Mobile Sensing app prototype screenshots presented to focus groups depicting 4 dashboards.

Our team developed a functional mobile app for Android cell phones that passively tracks behavior in patients with SMI. Using mobile sensors, phone use, and phone communication, the app collects data that are relevant to 3 behavioral domains: sleep, sociability, and activity. Phone sensors (eg, accelerometer sensors and ambient light sensors) collect data on location, movement, sound, and light; phone usage transmits data related to apps used and screen on-time; and phone communication transmits data on the number of calls and SMS text messages placed (not content). These input data are used to develop individualized estimates of the 3 behavioral domains previously listed. Figure 2 shows the digital dashboards of the app for the 3 behavioral domains by type, intensity, and over time.

### Data Collection

Qualitative data were collected from patients in treatment for SMI and clinicians providing mental health care (eg, psychiatrists, psychologists, nurses, and social workers) at 1 Veterans Administration (VA) medical facility and is summarized in Table 1. Qualitative researchers assisted in data collection instrument design, development of interview guides, transcript verification, and data coding and analysis. Interview and focus group guides were written using HITAM as a framework. The method of administering minimized biases by asking open-ended questions before more direct probes.

**Table 1:** Summary of data collection methods for patient and clinician attitudes toward mobile sensing.

Data collection method	Dates	Participants, n	Participants
Semistructured interviews	December 2020 to March 2021	16	Clinicians
Focus groups (n=3)	February to April 2021	17	Patients in treatment for SMI^a^
Usability tests	April to June 2021	8	Patients in treatment for SMI

^a^SMI: serious mental illness.

Semistructured interviews with 16 clinicians were conducted between December 2020 to March 2021. Interviews lasted approximately 30 minutes, were audio recorded, and transcribed with participant consent. Interviews first aimed to assess clinicians’ general interest and perspectives on using the Mobile Sensing app, its web-based clinical dashboard, and its data (eg, passive data monitoring patients’ sleep, activity, and sociability) in their clinical practice. Then, feasibility, acceptability, facilitators, barriers, and suggestions for patient self-care and for clinician use in clinical practice were discussed.

Three 45-minute focus groups of 4-6 patients (n=17 in total) in treatment for SMI were conducted between February and April 2021. Participants were recruited through in-person and web-based study recruitment presentations made at the Psychosocial Rehabilitation and Recovery Center and the Domiciliary Residential Rehabilitation Center, and through study flyers posted in the facility’s mental health clinic. Participants were paid US $20. Focus group discussions consisted of showing pictures of the Mobile Sensing app prototype (see Figure 2) and describing what dashboards, charts, and graphs showed them and their clinicians. They generated data on patients’ preferences, interests, and critical input to inform the intervention development.

Usability tests were conducted with patients in treatment for SMI between April and June 2021 (n=8). Participants were recruited from our focus groups. Each usability test lasted approximately 30 minutes and participants were paid US $20. Patients participated in usability tests individually using an Android phone provided by a study team member; tests concentrated on downloading, opening, reviewing the dashboard, and closing the app to track patient feedback to inform a more user-friendly app.

### Data Analysis

A team of 3 analysts conducted a directed content analysis [29] using a rapid qualitative analytic approach [30-32]. We began with a priori coding categories based on interview guides developed using HITAM measures. Emergent categories captured additional content relevant to patients with SMI and the VA health care system. Consistent with a rapid qualitative approach [30], team members reviewed interview notes, summarized, and validated information by category into tables to capture relevant content sorted by patients and clinicians. Information was synthesized across individual summaries, using constant comparison, to understand patient and clinician perceptions regarding the utility and acceptability of the smartphone app. This paper focuses on results regarding the utility and acceptability of a hypothetical mobile app that uses mobile sensing and a clinical dashboard.

## Results

### Clinician and Patient Characteristics

All clinicians who completed an interview (n=16) provided mental health care to patients with SMI. Further, 7 psychiatrists and 1 nurse provided clinical care, 3 psychologists provided psychosocial casework, and 5 social workers were case managers. Half of the clinicians worked primarily at a psychosocial rehabilitation and recovery center, while the others worked in a residential rehabilitation program or general medicine. Table 2 summarizes clinicians’ roles and the departments in which they work at the VA health care system.

Of the 21 patient participants recruited, 17 consented and participated in 1 of the 3 focus group discussions. All focus group participants (which includes the usability testers recruited from focus groups) were male; most were in-patients (n=13, 77%), and almost a quarter were outpatients (n=4, 23%). Demographically, focus group participants were diverse, comprised of a range of ages (median 45, range 21-66 years), an equal number of self-identifying non-Hispanic White and Black participants, 2 self-identifying Hispanic participants, and some who declined to respond to race or ethnic identity questions.

**Table 2:** Clinician characteristics.

Variable		Values, n (%)
**Role**		
	Psychiatrist	7 (44)
	Nurse	1 (6)
	Psychologist	3 (19)
	Social worker	5 (31)
**Department**		
	Residential rehabilitation program	3 (19)
	General medicine	2 (12)
	Homeless patient aligned care team	3 (19)
	Psychosocial rehabilitation and recover center	8 (50)

### Perceived Usefulness: Patient Perspectives Using Mobile Apps and Mobile Sensing

From focus groups with patients with SMI, we found that most reported that they continuously use apps on their smartphones for various purposes.

Every time you pick up your phone you use an application pretty much. I’d say probably about a fourth of the day.

focus group (FG1)

Oh yeah, I've got like four pages of 'em.

FG2

**Figure 3:**
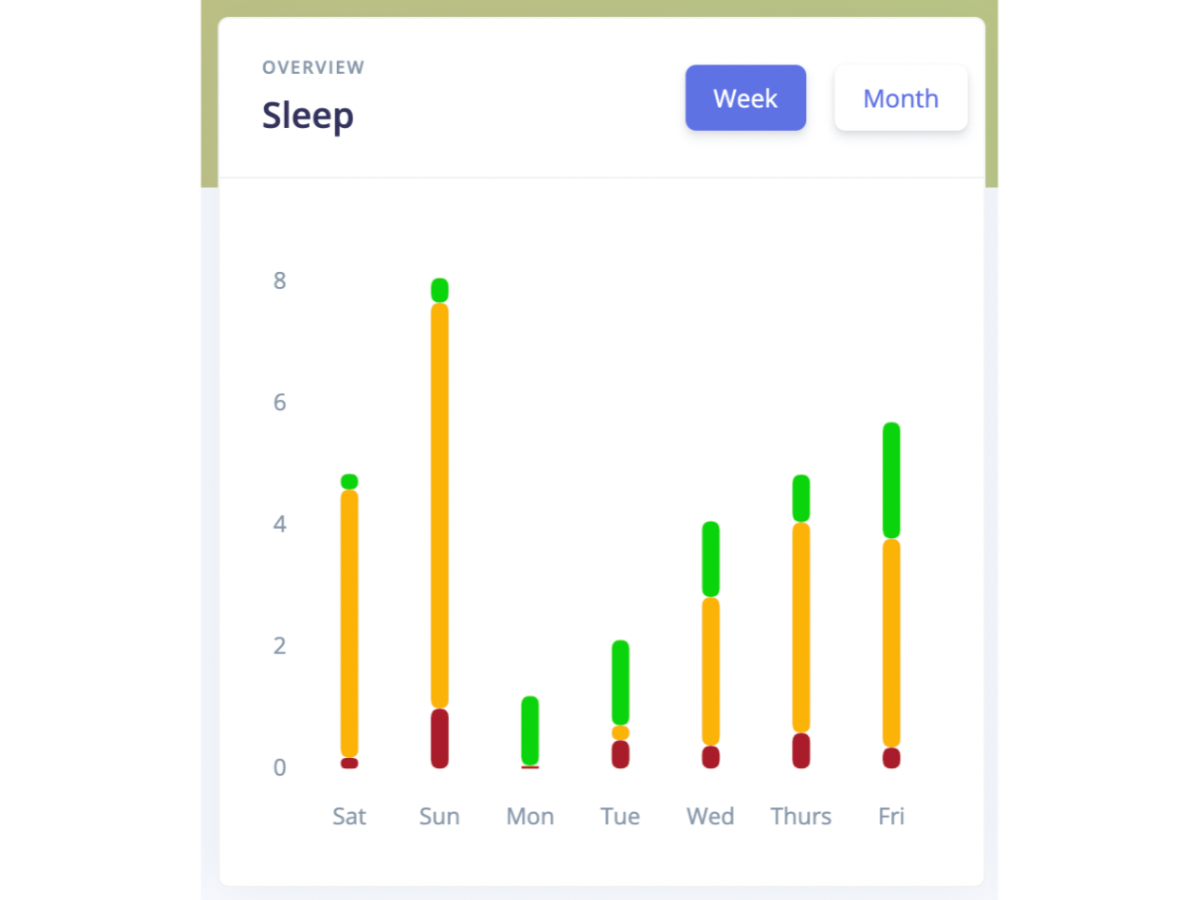
Mobile Sensing sleep dashboard (week view).

Most patients downloaded apps on their phones themselves; however, a few said that they had other people download apps for them or that their phones came with apps already downloaded. Participants reported that they used apps to access the following: music (eg, Spotify and iTunes), social media (eg, Instagram and YouTube), health (eg, MyHealtheVet, tracking steps, and managing cholesterol or weight), travel (eg, Waze), or fast-food restaurants (eg, McDonald’s and Taco Bell). Feedback about the Mobile Sensing app specifically (see Figure 3) was mostly positive. About half of patient participants stated that the Mobile Sensing app would be easier to use than most other apps and participants generally liked the idea of using the app for self-monitoring.

I think that’s a great idea, great concept. I think that’d be easier for people that wanna have their health monitored and that there isn’t much interaction that they have to do with the app; it’ll just run in the background.

FG1

I think that in a way, it’d be good for me, because it’ll keep me out of trouble, because I got to take my phone where I’m going. You know, I don’t wanna do ‘those things.’ It’ll make it stronger for me not to do 'em.

FG2

Yeah, I’d still use it. I’m not doing anything I shouldn’t do anymore, so...That's what turning over a good leaf will do for you.

FG3

As the above quotations illustrate, most patients were interested in apps with information that would help them self-monitor and manage their illness. Some commented that the Mobile Sensing app would be useful for “keeping out of trouble” as the app would encourage them to self-regulate. They said it would be easy to download onto their smartphones and to access and use the dashboards. Some found the app “innovative,” easy to read, and useful for self-monitoring their sleep.

### Perceived Usefulness and Perceived Ease of Use: Patient Perspectives About Clinicians, Dashboards, and Alerts

Patient perspectives about clinicians viewing dashboards and receiving alerts were mixed, but mostly positive (Textbox 1).

A few participants reported that the passive data would help clinicians gain better insights about their individual health status. A few also stated that the passive data would be more accurate than their self-reports. The advantages to using the app were that patients did not need to remember to turn it on, that it reports data automatically, and that it would be good to be contacted by a clinician if they noted significant changes in their behaviors. Concerns about passive monitoring included privacy factors, not understanding how it works or differentiates activities (sleep, walking, and driving) and where data goes and to whom, particularly the government “seeing” your social life. Other patient concerns were remembering to look at it and turning it off accidentally. Despite their concerns, participants stated that they would use the app if it were available to them as long as it did not drain their phone battery or use up data (*behavioral intention*).

**Patient perspectives about clinicians viewing dashboards and receiving alerts.**

**Positive aspects**

- *If this gives an alert to my doctor or facilitators, then they have brought the attention of my shortcomings that they contact me. That’s what I like. I also feel good about that type of monitoring. For instance, my Bank of America. If they see that my spending habit is different than my normal spending habits, they would immediately alert me.* [FG3]
- *This will let the VA know that hey, you know what? Maybe this guy needs to be checked on, because he hasn’t been doing anything. He’s hardly moved. He’s not on his cellphone. Let’s see what’s going on with this guy’s head.* [FG2]

**Negative aspects**

- *The con I would think would be [that] some people might say oh they’re taking my information down and stuff like that. Maybe some people they don’t like to be monitored.* [FG1]
- *Any GPS app will know exactly where you're at all the time [agreement]. But your phone does that anyway.* [FG2]

### Perceived Ease of Use: Patient Usability Tests

Findings from individual usability tests with individual testers to inform user-centered app design echo feedback we received during focus group discussions. Downloading and opening the Mobile Sensing app proved easy for smartphone users, with some expressing concerns about potential problems for older patients. Overall, patients with SMI did not have problems downloading, opening, closing, or understanding the app dashboards that the following quote exemplifies.

The graphs and charts tell when you are awake, light sleep, deep sleep. Not only does it have it on this little box, but when I click on Saturday, it tells, on the little red box, how many hours I was awake and 2.2 deep sleep. One thing I do like about it is that it shows the day that you are stuck on. The little box breaks it down even more. I like that it is consistent all the way through. Yes, I do find it easy to understand.

usability tester (UT3)

Most reported that graphs and numbers were clear and easy to understand, while a few were not initially sure what D (day), W (week), or M (month) signified. Testers made 2 key suggestions for improving the usability of the app, posting tutorials or demonstrations on the VA store web page, and optimizing font sizes for graphs and other graph components.

### Perceived Threat and Subjective Norm: Clinician Perspectives on Mobile Apps and Passive Monitoring

From interviews with clinicians, we found that most had no experience using mobile apps for mental health in their clinical practice and that half were unaware if their patients with SMI used mental health applications. According to clinicians, the idea of using mobile apps to passively monitor the health trends of patients with SMI was viewed as potentially advantageous but with notable caveats:

It will be a better way to support our client’s goals, especially the population that we are serving. There is no app that addresses SMI. I also think it will be a hard sell for older patients with little tech background; they will have a difficult time adapting to an app.

clinician (C4)

I see them as helpful tools as long as they are validated and there's no commercial influence.

C7

Information [from passive data visualized on dashboards] would be easy and more reliable than if they self-reported that information.

C13

Some clinicians felt that mobile apps were the “new direction of psychiatry,” that passive data were more feasible (eg, reliable) than active (eg, patient-reported) data, and that using the Mobile Sensing app may be a better way to support the SMI population by providing an added layer of clinical care in conjunction with normal care by helping them know more about patients’ health status, trends over time, and receiving alerts. However, clinicians expressed concerns about the clinical usefulness of the data and the need to tailor or individualize data to know if a patient was experiencing worsening symptoms.

About half of clinicians (n=7) anticipated challenges in “selling” the idea to their patients with SMI due to projected SMI patients’ paranoia or older patients’ reluctance to use technology. Clinicians perceived advantages of Mobile Sensing for patients were that patients did not have to actively participate, that they could “see evidence” to better understand their mental health, and that the app may motivate them to seek help sooner. Clinicians perceived disadvantages for patients were paranoia, not having or losing their smartphones, and potential technological limitations. A few clinicians wanted more information on how the passive data played out in real-life situations.

### Attitudes: Clinician Perspectives on Passive Data and Receiving Alerts

When asked about the usefulness of passive data, clinicians said that it may give them a better sense of what is going on in patients’ lives, since self-reports are subjective and hampered by recall bias.

I think it’s helpful because our admission rates are low, so it would be great receiving alerts for decompensation. My concern is monitoring social connection through the number of texts and not looking at content, which could be confounding (i.e., a delusional patient is making calls but they’re not in line with socialization).

C5

I see passive data as useful. It would be good to know how they’re behaving outside the clinic, because it is difficult to verify sleep, activities, etc.

C11

It would also be helpful to locate a Veteran if they are suicidal. Patients with challenging or complex issues could be worried about being tracked so it might be a harder sell, depending on what they focus on (i.e., delusional thoughts might focus on government or finances). It would be useful for those accustomed to technology.

C15

Over half of clinicians interviewed said that, if patients used it and they could establish a baseline, they would very likely review data from the app to monitor patients’ mental health trends over time. They felt that the app could be used as a reporting tool, similar to exercise apps that track steps, and that since the dashboard data seemed visually easy to review it may open up time during regular appointments to cover more with patients. Others felt the data would be useful for knowing when symptoms were ramping up and the need for medication adjustments.

Most clinicians found receiving alerts for worsening mental health symptoms useful, but with caveats.

Alerts would be helpful for huge caseloads; having it link to [medical records] and have a visualization (i.e., a dashboard) with a baseline for individual patients would be helpful. It would be a way to flag particularly concerning trends.

C5

A second person or second team to respond to alerts on weekends, after hours, or while someone was on vacation or leave.

C13

About half of the clinicians said that alerts would be useful for managing large caseloads and detecting major behavioral trends. There were a few concerns about receiving them, which focused on the responsibilities of responding to them outside of work hours and on weekends. Most felt that receiving alerts by secure email via electronic health records (Computerized Patient Record System, CPRS; GTI INFOTEL) or having the dashboard integrated into Computerized Patient Record System (CPRS) would work best so that they could respond appropriately, either by phoning the patient or scheduling an appointment through VA Video Connect (VA) software within 24 hours.

### Perceived Ease of Use: Clinician Perspectives on Uptake and Benefits to Care

Barriers to uptake of the Mobile Sensing app that clinicians discussed focused primarily on potential workload issues and alert fatigue.

I can envision the data becoming overwhelming for some clinicians who already feel overworked. The challenge will be to get the data to the clinicians so that it does not overwhelm them and simplify it as much as possible.

C4

Workload, especially the current workload. There should be more psychiatrists who share patient load if it takes more time to use.

C2

All clinicians discussed workflow and that monitoring alerts and follow-up time may add uncompensated work. Clinicians suggested having a second person or team to respond to alerts during weekends, evenings, and time off as well as automatic connections to crisis lines with VA in severe situations. They also discussed the potential cultural shift for patients, being treated based on data that they have not self-reported and the “big brotherish” nature of passive data collection. Despite these barriers, most clinicians said that they would recommend the Mobile Sensing app to their patients (*attitudes and behavioral intention*).

## Discussion

### Principal Findings

This study contributes to the literature regarding health IT acceptability among patients with SMI and mental health clinicians. Framed by the HITAM model, findings from our study indicated that patients and clinicians were receptive to remote, passive monitoring using smartphone technology to rapidly detect worsening mental health symptoms. Patients with SMI appeared interested in self-monitoring and having alerts sent to their mental health clinicians (*health concerns and perceived threats*). They found the app easy to navigate and dashboard graphics easy to understand (*perceived usefulness and ease of use*)*.* Clearly, our findings showed that clinicians lacked experience using apps in their clinical practice and using health IT in clinical care (*health concerns and* *perceived threats*). However, both patients and clinicians recognized the potential advantages of passive monitoring and receiving alerts for worsening symptoms (*HIT reliability and perceived usefulness*). Patients were concerned with privacy factors and data or battery issues, while clinicians were apprehensive about workload and alert fatigue (*HIT self-efficacy and perceived ease of use*). Despite these trepidations, most patients and clinicians stated that they would use the Mobile Sensing app if or when it was available (*behavioral intention*).

Our findings suggest that the quality of the app’s output, its reliability, and other factors, such as the health care environment and clinician experience using mobile technologies, are all significant considerations prior to implementation. While the US Department of Veterans Affairs offers a wide range of mental health–related apps [33], implementing a mobile digital tool for use by clinicians and patients is difficult [22,34,35]. It requires buy-in at the organizational and end user (clinician and patient) levels to achieve optimal outcomes [6,23,36,37]. While we found that almost all of the clinicians we interviewed had no experience using mobile technologies or health IT in their clinical practice at the time that they were interviewed, limited access to mental health services during COVID-19 facilitated the rapid development of digital clinics and mobile apps, affording clinicians exposure to and success with digital health [23,34,35,38]. Similar to these recent digital health studies, we found that it is critical to rethink the clinical workflow to successfully implement and integrate an app using mobile sensing. Additionally, due to the challenges of designing mobile technology for use by the SMI population [21], we integrated graphic design style and tutorial suggestions gleaned from patient focus groups and usability tests into the development of the Mobile Sensing app. Potential facilitation strategies for implementation could include using digital navigators for patients and educating frontline staff for clinical deployment.

Successful adoption of Mobile Sensing ultimately depends on the actual end users of the data, in this case, both patients with SMI and clinicians. The VA is moving toward a hybrid environment, especially since the pandemic. A hybrid environment drives app-based approaches to mental health care and creates challenges for recruiting patients. All these factors indicate the need for a plan to rollout and implement the project in a feasible way. The benefits of the Mobile Sensing app for patients include its function as a self-management support tool and an alert notification for behavioral changes. For clinicians, it provides a care coordination support tool that notifies them of changes in patients’ behaviors. A clinician will be able to contact a patient in a timely manner and alert the health care organization that someone needs a check-in for their mental health or other chronic conditions. However, clinicians also expressed reservations about time and workflow adjustments that using the Mobile Sensing dashboard may require of them. Despite these reservations, clinicians also acknowledge the assessment and treatment strategies being developed and deployed for treating mental health [6,39]

This work is limited by its restriction to 1 VA site and its generalizability to other populations (eg, female veterans and those outside the VA) and health care systems. The COVID-19 pandemic created challenges in conducting this research, leading to innovative data collection methods such as hybrid focus groups (ie, web-based and in-person) conducted outdoors, and may have limited our sample size and sample diversity. Alternative strategies may be needed for the most vulnerable patients with SMI such as low socioeconomic status populations with SMI who may not have consistent or any access to a smartphone.

### The Mobile Sensing Phase

This paper reported on phase 1 of the Mobile Sensing Study Design, User-Centered Design Phase. Recruitment and enrollment for the mobile sensing intervention phase (phase 2) began in October 2021 with the goal of recruiting 125 patients and is expected to conclude in July 2023. Qualitative and quantitative assessments during and after deployment of this phase will measure patient experiences as outcomes. Mobile phone sensor and utilization data will be used to develop individualized estimates of sociability, activity, and sleep that will also be measured through weekly interviews. Various machine learning algorithms will be used to build, train, and select prediction models for each patient’s behavioral assessment domains, and evaluated for predictive performance and cross-validation. Postsensing phase interviews will assess how to engage patients and reflect on findings, implementation issues, and resources needed for sustaining and incorporating mobile sensing with the Mobile Sensing app into routine practice.

### Conclusions

Many people with SMI would like to have mobile informatics tools that can support their illness and recovery. Similar to other populations, such as older adults or people experiencing homelessness, this population presents some challenges to adoption and implementation. HITAM provides a useful lens with which to analyze the acceptability and usability of mental health mobile apps. Health care organizations will need to provide resources to address these and support successful illness management among these populations. Clinicians are also supportive of technological approaches, with adapting to using informatics data in their workflow as the primary challenge. Despite clear challenges using technology-based assessments like mobile sensing, technological developments are exciting and increasingly designed to be acceptable to patients. The research development–clinical deployment gap will have to be addressed by health care systems, similar to the case for computerized cognitive training. It will be necessary to ensure clinicians operate at the top of their skill set and that they are not overwhelmed by administrative tasks, data summarization, or reviewing data that does not indicate a need for their intervention.

## Abbreviations

C: clinician
FG: focus group
HIT: health information technology
HITAM: Health Information Technology Acceptance Model
SMI: serious mental illness
VA: Veterans Administration
